# The changing demographics and severity in hospitalized patients across COVID-19 variants: A national cohort study

**DOI:** 10.1017/cts.2024.1166

**Published:** 2025-01-06

**Authors:** Priyanka Parajuli, Lara A.C. Phipps, Roy Sabo, Rasha Alsaadawi, Amanda Robinson, Evan French, Richard K. Sterling, N3C. consortium

**Affiliations:** 1 Department of Internal Medicine, Virginia Commonwealth University, Richmond, VA, USA; 2 University of North Carolina at Chapel Hill, Chapel Hill, NC, USA; 3 C. Kenneth and Dianne Wright Center for Clinical and Translational Research, Virginia Commonwealth University, Richmond, VA, USA; 4 Department of Biostatistics, Virginia Commonwealth University, Richmond, VA, USA; 5 Division of Gastroenterology, Hepatology, and Nutrition, Richmond, VA, USA; 6 Division of Infectious Disease, Virginia Commonwealth University, Richmond, VA, USA

**Keywords:** COVID-19 variants, demographics, survival, comorbidities, mechanical ventilation

## Abstract

**Introduction:**

The respiratory syndrome coronavirus (SARS-CoV-2) has undergone genetic evolution and led to variants of concern that vary in transmissibility and clinical severity.

**Methods:**

This retrospective cohort analysis studied 232,364 hospitalized COVID-19-positive patients in the National COVID Cohort Collaborative [April 27, 2020 and June 25, 2022]. The primary outcomes were to compare demographics and need for mechanical ventilation and 30-day mortality across variants including Alpha (B.1.1.7), Delta (B.1.617.2), and Omicron (B.1.1.529).

**Results:**

The severity of SARS-CoV-2 decreased in the omicron-subsequent wave with decreased utilization of mechanical ventilation and decreased 30-day mortality among patients with comorbidities like diabetes mellitus, obesity, and liver disease. Although with each subsequent wave, the sex distribution remained equal and constant, there was an increase in rates of diabetes, liver disease, and respiratory disease amongst patients hospitalized with COVID-19 over the COVID waves despite the decreasing 30-day mortality and mechanical ventilation.

**Conclusions:**

Despite changes in demographics over time, more recent COVID waves were associated with decreasing severity and mortality. These observations will help guide specific and effective resource allocation and patient care.

## Introduction

The severe acute respiratory syndrome coronavirus (SARS-CoV-2) has undergone genetic evolution and led to new variants of concern that vary in transmissibility and clinical severity [1]. Recent studies of the Omicron variant have shown reduced odds of hospitalization compared to the prior Delta variant [2–5]. However, prior analyses have not considered comorbidities. Among vaccinated individuals, omicron was also found to have 2.4–3.2 times higher transmissibility compared to the delta variant which was attributed to immune evasion [6]. Ex-vivo studies also showed that Omicron had less efficient replication in lung parenchyma. Hence, despite the higher transmissibility, newer variants may be less severed because of their lesser replication abilities in the lung. Healthcare disparities and the disproportionate impact of COVID-19 in relation to race and ethnicity have been validated including for mortality and hospitalization rates [7]. Underlying comorbidities including obesity, chronic obstructive lung disease (COPD), diabetes, liver disease, heart failure, and obesity have been outlined as risk factors for increased odds of requiring invasive ventilation and mortality [8–19]. Due to the evolving nature of SARS-CoV-2, it is useful to evaluate the demographics and need for mechanical ventilation and 30-day mortality with the COVID-19 variants Alpha, Delta, and Omicron. Due to increased vaccination efforts and increased protection from previous infection against re-infection, we hypothesized that the omicron variant (B.1.1.529) would show decreased clinical severity adjusted for comorbidities.

## Methods

### Patient characteristics

**Figure 1.**
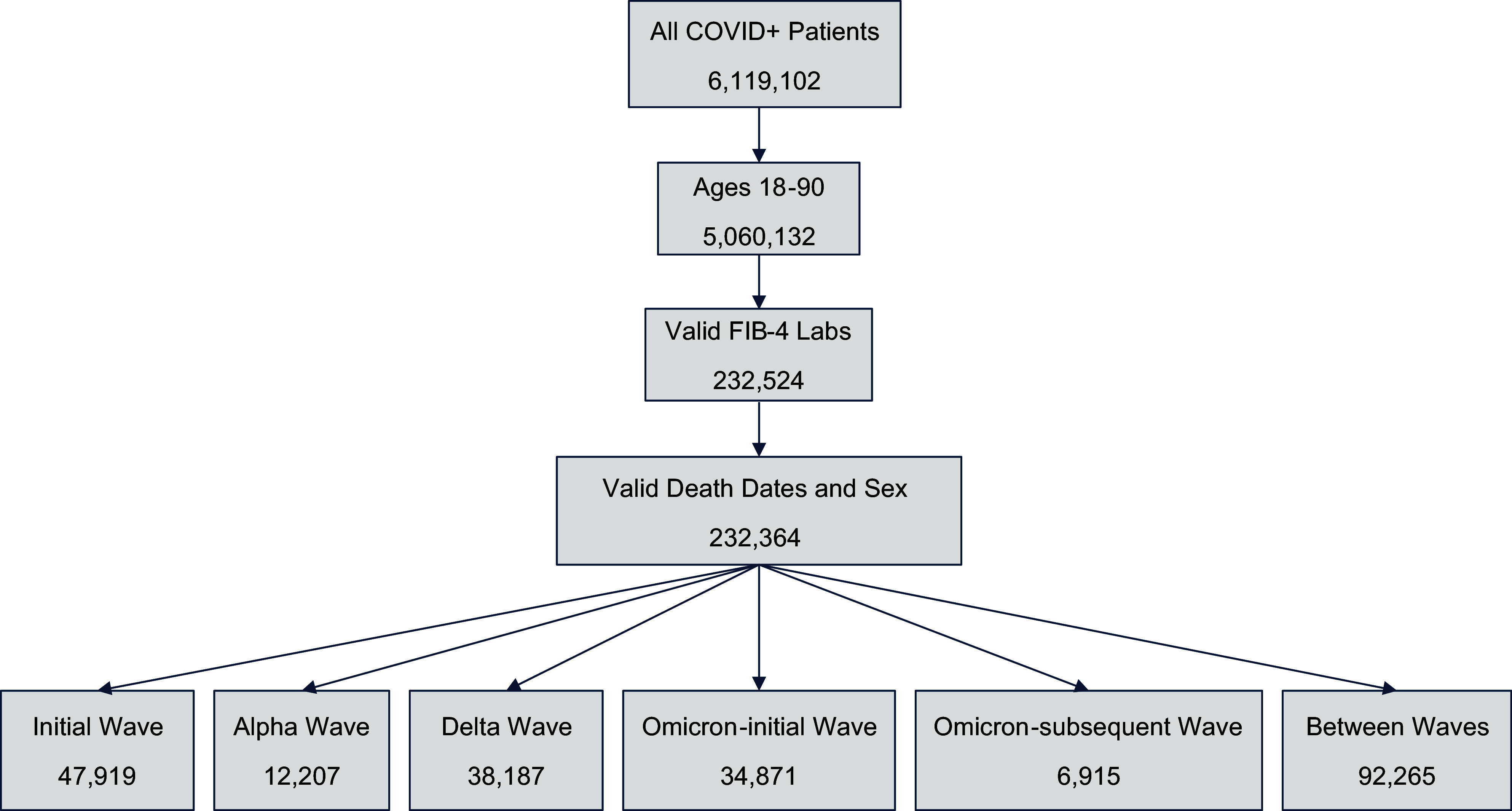
Breakdown of the sample size of COVID-19 positive patients by age, valid FIB-4 levels, and valid death dates. Abbreviations: Fibrosis-4 (FIB-4).

This is a population-based retrospective cohort analysis of COVID-19 patients in the United States using the National COVID Cohort Collaborative (N3C) [8,20]. N3C uses a standard structure to harmonize datasets into a common data model (https://covid.cd2h.org/workstreams/harmonization/). Fig. 1 shows a breakdown of the sample size utilized in this study. A sample size of 232,524 patients who tested positive for COVID-19 between April 27, 2020 and June 25, 2022 with sufficient data to calculate BMI was collected, as described previously [8]. Of those, 47,919 were hospitalized during the initial COVID-19 wave, 12,207 during the Alpha wave, 38,187 during the Delta wave, 34,871 during the Omicron-initial wave, and 6,915 during the Omicron-subsequent wave. COVID-19 waves (Table 1) were defined using date ranges, and waves were not contiguous to allow time for the prevalent variant to shift. Inclusion was restricted to patients between the ages of 18 and 90, as well as to those with measurements for aspartate aminotransferase, alanine aminotransferase and platelet measurements – collectively the components of the fibrosis-4 index (FIB-4) – taken within 24 hours of hospitalization as part of a separate study of COVID-19 variants to help identify those with liver disease either undiagnosed or not recorded [8,21]. Individual patient demographic and clinical characteristics were summarized overall and by COVID wave. Frequencies and percentages were reported for categorical variables, while means and standard deviations were reported for continuous variables.

**Table 1.** Date ranges of COVID-19 variants

Variant	Start date	End date
Initial	April 27, 2020	November 23, 2020
Alpha	March 29, 2021	May 24, 2021
Delta	August 2, 2021	December 6, 2021
Initial Omicron	December 20, 2021	March 14, 2022
Subsequent Omicron	March 28, 2022	June 25, 2022
Overall	April 27, 2020	June 25, 2022

### Statistical analysis

We fit two sets of logistic regression models: one set with mechanical ventilation use as the outcome and the other set with 30-day mortality; note for both outcomes we model data from each variant wave separately. We fit univariable models adjusting for individual patient characteristics including sex race/ethnicity; diabetes; comorbid cardiac, liver (by diagnosis code or increased FIB-4), and respiratory disease; obesity; days in the hospital; admission to ICU within 30 days of COVID diagnosis; FIB-4; and treatment directed against SARS-CoV-2. Antivirals with potential activity against SARS-CoV-2 evolved over the waves and included dexamethasone, remdesivir, lopinavir/ritonavir, baricitinib, casirivimab with imdevimab, etesevimab, molnupiravir, nirmatrelvir/ritonavir, and sotrovimab. We also fit multivariable logistic regression models for both outcomes adjusting for all patient characteristics in conjunction. We did not include age in the multivariable models as it is included in the formula for FIB-4. We limited our models to one degree of freedom for every 10-20 events in the data set [22].

For ethnic populations of American Indian and Native Hawaiian races who had fewer than 20 counts, data were categorized as “other” to obfuscate the results. We chose to combine these groups when presenting summary statistics to avoid potentially identifying these patients. We were still interested in understanding patterns of mechanical ventilation and mortality within these groups, so we included the more granular categories in the models. Linearity was assessed for all continuous covariates, while variance inflation factors were used to assess collinearity. All summaries and analyses were obtained using the R statistical computing software within the N3C cloud computing environment.

## Results

### Demographics and clinical characteristics

Subject characteristics by wave are shown in Table 2. Mean patient age was 60 years overall. Throughout the variants, the data showed relatively equal numbers of male and female patients. Comorbid diseases were common across the COVID waves: cardiac disease in 33%, diabetes in 35%, liver disease in 11%, respiratory disease in 18%, and between 45 and 51% of patients were obese. Interestingly, there was an increase in cardiac, respiratory, and liver disease amongst patients with more recent variants. Overall, the majority (56%) of patients were non-Hispanic White and nearly 20% of patients were non-Hispanic Black or African American. Roughly 14% of patients were Hispanic or Latino overall, ranging from 7% during the subsequent Omicron wave to 20% during the initial wave.

**Table 2.** Patient demographics and clinical characteristics by COVID variants

Characteristic	Value	Initial (n,%)	Alpha (n,%)	Delta (n,%)	Omicron-initial (n,%)	Omicron- subsequent (n,%)	Overall (n,%)
Age (years)	Mean (SD)	60.39 (17.08)	56.52 (16.76)	58.17 (16.99)	60.14 (17.91)	62.93 (18.91)	60.48 (17.19)
Sex	Male	25,422 (53.05 %)	6010 (49.23 %)	19,859 (52.00 %)	17,888 (51.30 %)	3431 (49.62 %)	122146 (52.57 %)
	Female	22,497 (46.95 %)	6197 (50.77 %)	18,328 (48.00 %)	16,983 (48.70 %)	3484 (50.38 %)	110218 (47.43 %)
Cardiac disease	Yes	14,369 (29.99 %)	3192 (26.15 %)	11,565 (30.29 %)	14,653 (42.02 %)	3304 (47.78 %)	77,241 (33.24 %)
	No	33,550 (70.01 %)	9015 (73.85 %)	26,622 (69.71 %)	20,218 (57.98 %)	3611 (52.22 %)	155123 (66.76 %)
Diabetes	Yes	16,712 (34.88 %)	3702 (30.33 %)	12,113 (31.72 %)	12,861 (36.88 %)	2373 (34.32 %)	80,547 (34.66 %)
	No	31,207 (65.12 %)	8505 (69.67 %)	26,074 (68.28 %)	22,010 (63.12 %)	4542 (65.68 %)	151817 (65.34 %)
Liver disease	Yes	4484 (9.36 %)	1188 (9.73 %)	4112 (10.77 %)	5255 (15.07 %)	1043 (15.08 %)	25,831 (11.12 %)
	No	43,435 (90.64 %)	11,019 (90.27 %)	34,075 (89.23 %)	29,616 (84.93 %)	5872 (84.92 %)	206533 (88.88 %)
Respiratory disease	Yes	7534 (15.72 %)	1831 (15.00 %)	6652 (17.42 %)	8300 (23.80 %)	1764 (25.51 %)	41,711 (17.95 %)
	No	40,385 (84.28 %)	10,376 (85.00 %)	31,535 (82.58 %)	26,571 (76.20 %)	5151 (74.49 %)	190653 (82.05 %)
Ventilator	Yes	4759 (9.93 %)	975 (7.99 %)	4487 (11.75 %)	3067 (8.80 %)	395 (5.71 %)	23,663 (10.18 %)
	No	43,160 (90.07 %)	11,232 (92.01 %)	33,700 (88.25 %)	31,804 (91.20 %)	6520 (94.29 %)	208701 (89.82 %)
Death in hospital	Yes	4327 (9.03 %)	798 (6.54 %)	4296 (11.25 %)	2911 (8.35 %)	267 (3.86 %)	22,262 (9.58 %)
	No	43,592 (90.97 %)	11,409 (93.46 %)	33,891 (88.75 %)	31,960 (91.65 %)	6648 (96.14 %)	210102 (90.42 %)
Treatment^§^	Yes	14,950 (31.20 %)	5413 (44.34 %)	19,886 (52.08 %)	14,446 (41.43 %)	3034 (43.88 %)	91,147 (39.23 %)
	No	32,969 (68.80 %)	6794 (55.66 %)	18,301 (47.92 %)	20,425 (58.57 %)	3881 (56.12 %)	141217 (60.77 %)
Race/ethnicity	White, NH	24,035 (50.16 %)	6186 (50.68 %)	25,702 (67.31 %)	21,108 (60.53 %)	4610 (66.67 %)	130536 (56.18 %)
	Black (NH)/AA	9568 (19.97 %)	3172 (25.99 %)	6463 (16.92 %)	7407 (21.24 %)	1072 (15.50 %)	45,363 (19.52 %)
	Hispanic or Latino	9791 (20.43 %)	1555 (12.74 %)	3383 (8.86 %)	3351 (9.61 %)	504 (7.29 %)	32,717 (14.08 %)
	Unknown	2563 (5.35 %)	810 (6.64 %)	1718 (4.50 %)	1850 (5.31 %)	441 (6.38 %)	14,462 (6.22 %)
	Asian (NH)	1253 (2.61 %)	345 (2.83 %)	446 (1.17 %)	715 (2.05 %)	225 (3.25 %)	6448 (2.77 %)
	Other (NH)	709 (1.48 %)	139 (1.14 %)	475 (1.24 %)	440 (1.26 %)	63 (0.91 %)	2838 (1.22 %)
Obesity	Yes	22,525 (47.01 %)	6266 (51.33 %)	17,546 (45.95 %)	15,783 (45.26 %)	3066 (44.34 %)	107921 (46.44 %)
	No	25,394 (52.99 %)	5941 (48.67 %)	20,641 (54.05 %)	19,088 (54.74 %)	3849 (55.66 %)	124443 (53.56 %)

Abbreviations: Non-Hispanic (NH), African American (AA), Pacific Islander (PSI), Body Mass Index (BMI).

*Proportion too small to quantitatively report.

^§^Treatment included agents directed at SARS-CoV-2 as defined in the methods.

### Mechanical ventilation utilization

The rate of mechanical ventilation utilization was 10% overall and varied across COVID waves with 10% during the initial wave, 8% during Alpha, 12% during Delta, 9% during Omicron-initial wave, and decreased further to 6% during the Omicron-subsequent wave. Table 3 summarizes univariate and multivariable results from logistic regression models with the odds ratios for mechanical ventilation of various comorbid diseases and demographics by COVID wave. Comorbid diabetes had higher odds of mechanical ventilation needs compared to those without diabetes across the initial, alpha, and delta variants in the unadjusted and adjusted models. However, it did not have any significant increase in odds in the omicron variates in the adjusted model. Patients with obesity (BMI ≥ 30 kg/m^2^) had higher odds of mechanical ventilation compared to non-obese patients across all waves in both unadjusted and adjusted models (1.46 [1.41, 1.51]) except in the Omicron subsequent wave, where no significant difference was observed. Comorbid liver disease had significantly higher odds of mechanical ventilation compared to those without across all COVID waves in the unadjusted and adjusted models except for omicron-subsequent which showed no significance in the adjusted model. The OR gradually decreased with the newer variates and overall, liver disease showed an elevated OR of 1.54 [1.47, 1.61]. In the adjusted models, patients experiencing comorbid cardiac disease had lower odds of lung ventilation compared to those without in all waves except Alpha, where no difference was observed. Patients with respiratory disease were associated with lower odds of mechanical ventilation during the Delta wave in the adjusted model with an OR [95% CI] of 0.85 [0.82, 0.88], while the association was not significant in the initial, Alpha, and Omicron waves. There was also an increased odds of mechanical ventilation with length of hospital stay across all COVID waves. There was also an increased odds of mechanical ventilation among patients admitted to the ICU within 30 days of COVID-19 diagnosis across all variants in both unadjusted and adjusted models.

**Table 3.** Odds ratios of mechanical ventilation using simple vs. Multiple logistic regression models for Alpha, Delta, omicron-initial, and omicron-subsequent waves

		Alpha		Delta		Omicron-initial		Omicron-subsequent	
Wave		SLR	MLR	SLR	MLR	SLR	MLR	SLR	MLR
Variables		OR (95% CI)	OR (95% CI)	OR (95% CI)	OR (95% CI)	OR (95% CI)	OR (95% CI)	OR (95% CI)	OR (95% CI)
Intercept		--	0.01 (0.01, 0.01)	--	--	0.01 (0.01, 0.01)	0.02 (0.01, 0.02)	--	0.02 (0.02, 0.03)
Female (sex)		0.79 (0.70, 0.90)	0.96 (0.82, 1.12)	0.75 (0.71, 0.80)	0.67 (0.65, 0.69)	0.74 (0.72, 0.77)	0.69 (0.64, 0.76)	0.06 (0.05, 0.07)	0.73 (0.59, 0.91)
Age*		1.04 (1.00, 1.08)	--	0.98 (0.96, 1.00)	1.00 (0.99, 1.01)	--	--	0.93 (0.89, 0.98)	--
Race^	American Indian or Alaska Native (NH)	1.48 (0.52, 4.23)	1.06 (0.32, 3.48)	2.20 (1.46, 3.31)	2.13 (1.77, 2.58)	1.52 (1.21, 1.90)	1.78 (0.99, 3.20)	0.90 (0.12, 6.77)	0.55 (0.06, 5.28)
	Asian (NH)	1.54 (1.10, 2.16)	1.51 (1.01, 2.25)	1.73 (1.36, 2.21)	1.52 (1.42, 1.64)	1.51 (1.39, 1.65)	1.39 (1.07, 1.82	1.08 (0.62, 1.87)	1.16 (0.63, 2.12)
	Black (NH)/AA	0.83 (0.71, 0.98)	0.73 (0.60, 0.89)	0.96 (0.88, 1.05)	1.09 (1.05, 1.13)	1.01 (0.97, 1.06)	0.99 (0.89, 1.10)	0.89 (0.66, 1.20)	0.91 (0.66, 1.25)
	Native Hawaiian, PSL	1.59 (0.36, 7.02)	2.17 (0.43, 10.83)	0.73 (0.26, 2.05)	1.34 (0.96, 1.88)	1.25 (0.85, 1.84)	1.58 (0.54, 4.58)	0 (0, inf)	0 (0, inf)
	Hispanic or Latino	1.02 (0.83, 1.24)	0.79 (0.62, 1.02)	1.18 (1.06, 1.32)	1.37 (1.32, 1.42)	1.28 (1.22, 1.34)	1.18 (1.02, 1.36)	0.81 (0.53, 1.24)	1.00 (0.64, 1.57)
	Other (NH)	0.80 (0.35, 1.85)	1.09 (0.42, 2.82)	0.78 (0.52, 1.16)	0.94 (0.79, 1.10)	1.11 (0.92, 1.34)	1.54 (1.02, 2.32)	1.47 (0.45, 4.83)	1.59 (0.42, 5.93)
	Unknown	0.89 (0.67, 1.18)	0.76 (0.54, 1.07)	1.14 (0.99, 1.32)	1.21 (1.14, 1.28)	1.21 (1.13, 1.29)	1.04 (0.86, 1.27)	1.14 (0.77, 1.69)	1.37 (0.90, 2.08)
Cardiac disease		1.19 (1.03, 1.37)	0.91 (0.77, 1.09)	0.82 (0.76, 0.88)	0.86 (0.84, 0.89)	0.71 (0.68, 0.73)	0.73 (0.67, 0.80)	0.87 (0.71, 1.07)	0.77 (0.61, 0.97)
Diabetes		1.63 (1.43, 1.87)	1.26 (1.06, 1.49)	1.46 (1.37, 1.55)	1.56 (1.52, 1.60)	1.28 (1.24, 1.33)	1.03 (0.94, 1.13)	1.10 (0.89, 1.36)	1.00 (0.79, 1.28)
Liver disease		2.56 (2.16, 3.03)	2.01 (1.63, 2.49)	1.83 (1.68, 1.99)	1.89 (1.82, 1.96)	1.54 (1.47, 1.61)	1.46 (1.31, 1.62)	1.39 (1.08, 1.80)	1.11 (0.83, 1.49)
Respiratory disease		1.11 (0.93, 1.33)	1.04 (0.83, 1.30)	0.91 (0.84, 0.99)	0.85 (0.82, 0.88)	0.90 (0.86, 0.95)	0.90 (0.81, 1.00)	0.85 (0.67, 1.09)	0.92 (0.70, 1.22)
BMI≥30		1.66 (1.45, 1.90)	1.37 (1.16, 1.61)	2.09 (1.96, 2.23)	1.74 (1.60, 1.88)	1.59 (1.48, 1.71)	1.43 (1.31, 1.56)	1.03 (0.84, 1.27)	1.11 (0.88, 1.40)
Days in hospital		1.10 (1.09, 1.10)	1.09 (1.09, 1.10)	1.11 (1.10, 1.11)	1.10 (1.09, 1.10)	1.08 (1.08, 1.08)	1.07 (1.07, 1.08)	1.07 (1.06, 1.08)	1.07 (1.06, 1.08)
Treatment^§^		2.75 (2.58, 2.92)	2.19 (2.04, 2.35)	1.52 (1.46, 1.59)	1.33 (1.27, 1.40)	Treatment	n/a	2.75 (2.58, 2.92)	2.19 (2.04, 2.35)

Abbreviations: Simple Logistic Regression (SLR), Multiple Logistic Regression (MLR), Body Mass Index (BMI), Alanine aminotransferase (AST), Aspartate aminotransferase (AST), Platelets (PLT), Not available (n/a).

*Odds ratios for 10-unit difference.

^Odds ratios for one unit increase in FIB-4.

^1^The reference group is White.

^§.^Treatment included agents directed at SARS-CoV-2 as defined in the methods.

Native Americans showed significantly higher odds of mechanical ventilation use during the initial wave, with an increased odds of mechanical ventilation at 1.91 [1.23, 2.95] and during the delta variant with an OR of 2.13 [1.77, 2.58] in the adjusted model. For Asians, there was an increased odds of mechanical ventilation use in the Alpha, Delta, and Omicron-initial variants, and no significant difference in the Omicron-subsequent in the unadjusted model. For Black and African Americans, there was an increase in odds of mechanical ventilation in the adjusted model for the Delta variant and in the initial variant and no significant difference in odds of mechanical ventilation in any Alpha or Omicron-subsequent in the adjusted model. Native Hawaiians did not show a significant difference in odds between all variants. Hispanic/Latino patients showed increased odds of mechanical ventilation during the initial variant, an increased OR of 1.37 [1.32, 1.42] in the adjusted model during the delta variant in the adjusted model, an increased odds of 1.18 [1.02, 1.36] during the Omicron-initial variant in the adjusted model.

### Associations with 30-day mortality

A sample of 25,250 patient deaths were identified within 30 days of hospitalization, 4,948 during the initial wave, 858 during the Alpha wave, 4,697 during the Delta wave, 3,583 during the Omicron-initial wave, and 374 during the Omicron-subsequent wave. The mortality rate within 30 days of diagnosis across all waves was 10%, with the highest rate (11%) observed during the Delta wave and the lowest (4%) during the subsequent Omicron wave. Table 4 summarizes odds ratios for 30-day mortality using simple and multiple logistic regression models for each specific variant. Comorbid diabetes was associated with higher odds of mortality (1.43 [1.39, 1.46]) in the unadjusted model for all waves. The models adjusting only for diabetes using data from each wave individually showed a similar association, with the exception of the initial wave and the Omicron-subsequent wave. During these two waves, diabetic patients had the same odds of death in the hospital within 30 days as non-diabetic patients. After adjusting for other factors, the odds ratio for comorbid diabetes was 1.31 [1.27, 1.35. When modeled alone across all waves, BMI greater than or equal to 30 kg/m^2^ was associated with decreased mortality odds (0.95 [0.92, 0.97]). While there were no differences in unadjusted mortality odds based on BMI in the Alpha, initial Omicron, or subsequent Omicron waves, the Delta wave was the only time period where higher BMI was associated with higher mortality odds (1.21 [1.14, 1.29]). In the adjusted model, obese patients had lower mortality risk than non-obese patients across all waves.

**Table 4.** Odds ratios for 30-day mortality using simple vs. Multiple logistic regression models for the initial, Alpha, Delta, omicron-initial, and omicron-subsequent waves

		Initial		Alpha		Delta		Omicron Initial		Omicron Subsequent		All Waves	
Wave		SR	MR	SLR	MLR	SR	MR	SR	MR	SR	MR	SR	MR
Variables		OR (95% CI)	OR (95% CI)	OR (95% CI)	OR (95% CI)	OR (95% CI)	OR (95% CI)	OR (95% CI)	OR (95% CI)	OR (95% CI)	OR (95% CI)	OR (95% CI)	OR (95% CI)
Male		1.36 (1.28, 1.44)	1.16 (1.09, 1.24)	1.31 (1.14, 1.51)	1.15 (1.00, 1.34)	1.25 (1.18, 1.33)	1.14 (1.07, 1.22)	1.39 (1.30, 1.49)	1.21 (1.12, 1.30)	1.48 (1.20, 1.83)	1.26 (1.01, 1.57)	1.34 (1.31, 1.38)	1.18 (1.15, 1.22)
Race	Native American	1.37 (0.91, 2.00)	1.23 (0.79, 1.85)	1.47 (0.44, 3.75)	1.48 (0.42, 3.95)	0.92 (0.53, 1.49)	0.71 (0.39, 1.21)	1.74 (1.03, 2.81)	1.51 (0.86, 2.53)	0.87 (0.05, 4.23)	0.63 (0.03, 3.69)	1.10 (0.88, 1.35)	0.96 (0.76, 1.19)
	Asian	0.71 (0.59, 0.86)	0.69 (0.56, 0.85)	1.21 (0.82, 1.72)	1.11 (0.74, 1.61)	0.97 (0.73, 1.27)	0.92 (0.68, 1.23)	0.87 (0.68, 1.10)	0.75 (0.58, 0.97)	0.80 (0.41, 1.42)	0.76 (0.38, 1.37)	0.97 (0.90, 1.04)	0.91 (0.84, 0.99)
	Black	0.66 (0.60, 0.71)	0.66 (0.61, 0.72)	0.68 (0.57, 0.81)	0.69 (0.57, 0.83)	0.64 (0.59, 0.71)	0.66 (0.59, 0.72)	0.58 (0.53, 0.64)	0.59 (0.53, 0.65)	0.67 (0.48, 0.92)	0.70 (0.50, 0.97)	0.67 (0.65, 0.70)	0.67 (0.65, 0.70)
	Native Hawaiian	0.80 (0.37, 1.51)	0.82 (0.37, 1.60)	1.58 (0.25, 5.67)	2.14 (0.33, 8.08)	0.29 (0.05, 0.94)	0.33 (0.05, 1.13)	0.86 (0.26, 2.15)	1.04 (0.31, 2.64)	2.23 (0.12, 12.62)	2.79 (0.15, 16.16)	0.80 (0.54, 1.13)	0.87 (0.58, 1.25)
	Hispanic	0.50 (0.46, 0.55)	0.60 (0.55, 0.66)	0.51 (0.39, 0.65)	0.58 (0.44, 0.75)	0.68 (0.61, 0.77)	0.78 (0.69, 0.89)	0.67 (0.59, 0.76)	0.76 (0.66, 0.86)	0.48 (0.27, 0.79)	0.58 (0.32, 0.96)	0.65 (0.63, 0.68)	0.75 (0.72, 0.78)
	Other	0.87 (0.64, 1.16)	0.96 (0.69, 1.31)	0.94 (0.39, 1.91)	1.02 (0.41, 2.20)	0.95 (0.66, 1.32)	1.29 (0.89, 1.82)	0.65 (0.42, 0.97)	0.84 (0.54, 1.27)	0.00 (0.00, 0.22)	0.00 (0.00, 0.23)	0.80 (0.68, 0.93)	0.93 (0.79, 1.09)
	Unknown	0.69 (0.60, 0.79)	0.77 (0.66, 0.89)	0.65 (0.47, 0.88)	0.64 (0.45, 0.88)	0.78 (0.66, 0.91)	0.84 (0.71, 0.99)	0.80 (0.68, 0.93)	0.88 (0.74, 1.03)	0.94 (0.60, 1.40)	1.09 (0.69, 1.65)	0.85 (0.81, 0.90)	0.90 (0.85, 0.96)
ICU admission		3.34 (3.01, 3.71)	2.64 (2.36, 2.96)	3.73 (2.84, 4.84)	2.66 (1.97, 3.55)	4.17 (3.74, 4.65)	3.07 (2.73, 3.46)	3.84 (3.39, 4.35)	2.95 (2.57, 3.37)	3.77 (2.64, 5.27)	2.97 (2.03, 4.27)	3.52 (3.35, 3.69)	2.78 (2.63, 2.93)
Cardiac disease		1.88 (1.77, 2.00)	1.47 (1.37, 1.57)	1.99 (1.72, 2.29)	1.49 (1.26, 1.76)	1.66 (1.56, 1.77)	1.32 (1.23, 1.42)	1.42 (1.32, 1.52)	1.29 (1.19, 1.39)	1.56 (1.27, 1.93)	1.46 (1.15, 1.84)	1.61 (1.56, 1.65)	1.35 (1.31, 1.39)
Diabetes		1.38 (1.30, 1.46)	1.27 (1.19, 1.36)	1.62 (1.40, 1.86)	1.37 (1.17, 1.60)	1.62 (1.52, 1.72)	1.39 (1.30, 1.49)	1.33 (1.24, 1.43)	1.26 (1.17, 1.36)	1.17 (0.94, 1.45)	1.10 (0.87, 1.38)	1.43 (1.39, 1.46)	1.31 (1.27, 1.35)
Liver disease		1.19 (1.08, 1.31)	0.75 (0.67, 0.83)	1.55 (1.26, 1.89)	0.93 (0.74, 1.16)	1.40 (1.28, 1.54)	0.94 (0.85, 1.04)	1.23 (1.12, 1.35)	0.88 (0.80, 0.97)	1.37 (1.04, 1.77)	1.02 (0.76, 1.35)	1.26 (1.21, 1.30)	0.84 (0.80, 0.87)
Resp. disease		1.37 (1.27, 1.47)	1.12 (1.03, 1.22)	1.38 (1.16, 1.65)	1.13 (0.92, 1.37)	1.34 (1.25, 1.45)	1.12 (1.03, 1.22)	1.05 (0.97, 1.14)	0.94 (0.86, 1.03)	0.94 (0.73, 1.19)	0.84 (0.64, 1.08)	1.20 (1.16, 1.24)	1.05 (1.01, 1.09)
BMI≥30		0.85 (0.80, 0.91)	0.76 (0.71, 0.81)	0.98 (0.86, 1.13)	0.89 (0.76, 1.04)	1.21 (1.14, 1.29)	1.00 (0.93, 1.07)	0.95 (0.88, 1.02)	0.83 (0.77, 0.90)	0.87 (0.71, 1.08)	0.77 (0.61, 0.97)	0.95 (0.92, 0.97)	0.85 (0.83, 0.88)
Days in hospital		1.05 (1.05, 1.05)	1.04 (1.03, 1.04)	1.07 (1.06, 1.07)	1.05 (1.05, 1.06)	1.05 (1.05, 1.06)	1.04 (1.04, 1.05)	1.05 (1.04, 1.05)	1.03 (1.03, 1.04)	1.05 (1.04, 1.06)	1.04 (1.02, 1.05)	1.05 (1.05, 1.05)	1.04 (1.04, 1.04)
Treatment^§^		1.36 (1.28, 1.44)	1.11 (1.04, 1.19)	1.08 (0.94, 1.24)	0.90 (0.77, 1.04)	1.45 (1.36, 1.54)	1.14 (1.07, 1.22)	1.60 (1.50, 1.72)	1.33 (1.23, 1.43)	1.34 (1.09, 1.65)	1.16 (0.93, 1.44)	1.26 (1.23, 1.29)	1.06 (1.03, 1.09)
FIB-4		1.25 (1.24, 1.26)	1.22 (1.21, 1.23)	1.25 (1.22, 1.28)	1.21 (1.18, 1.24)	1.22 (1.21, 1.24)	1.20 (1.19, 1.21)	1.20 (1.19, 1.22)	1.19 (1.18, 1.20)	1.20 (1.16, 1.23)	1.18 (1.14, 1.21)	1.23 (1.22, 1.23)	1.21 (1.20, 1.21)

Abbreviations: Simple Logistic Regression (SR) Modeling, Multiple Logistic Regression (MR) Modeling, Intensive Care Unit (ICU), Body Mass Index (BMI).

^§^Treatment included agents directed at SARS-CoV-2 as defined in the methods.

Comorbid liver disease was associated with higher odds of mortality (1.26 [1.21, 1.30]) in the unadjusted model, and this association was observed in the model for each wave. In the adjusted model for all waves, comorbid liver disease was associated with lower mortality odds (0.84 [0.80, 0.87]), as well as during the initial wave (0.75 [0.67, 0.83]) and the initial Omicron wave (0.88 [0.80, 0.97]). There was no difference in adjusted mortality odds based on liver disease diagnosis in the other waves. Comorbid cardiac disease was associated with increased odds of mortality in the unadjusted model for all waves (1.61 [1.56, 1.65]), and this association was observed in the model for each wave. After adjusting for other factors, comorbid cardiac disease remained significantly associated with increased mortality across all waves (1.35 [1.31, 1.39]) as well as within individual waves. Comorbid respiratory disease was associated with increased mortality odds across all waves in the unadjusted model (1.20 [1.16, 1.24]), as well as during the initial (1.37 [1.27, 1.47]), Alpha (1.38 [1.16, 1.65]), and Delta waves (1.34 [1.25, 1.45]). There was no difference in the unadjusted odds of mortality during either Omicron wave. After adjusting for other factors, comorbid respiratory disease was still associated with increased mortality across all waves (1.05 [1.01, 1.09]), as well as during the initial (1.12 [1.03, 1.22]) and Delta waves (1.12 [1.03, 1.22]).

Admittance to the ICU within 30 days of COVID diagnosis was associated with higher mortality odds across all waves in the unadjusted model (3.52 [3.35, 3.69]), as well as during all waves individually. ICU admittance remained associated with higher mortality odds after adjusting for other factors across all waves (2.78 [2.63, 2.93]), as well as within each wave individually. The odds of mortality increased with length of stay across all waves in both the unadjusted (1.05 [1.05, 1.05]) and adjusted models (1.04 [1.04, 1.04]), as well as within individual waves. Treatment within 30 days of COVID diagnosis was associated with increased mortality odds across all waves in the unadjusted (1.26 [1.23, 1.29]) and adjusted models (1.06 [1.03, 1.09]), as well as during the initial, Delta, and initial Omicron waves. There was no difference in mortality based on treatment status in the other waves.

White patients experienced higher mortality odds compared to Black and African American patients (0.67 [0.65, 0.70]), Hispanic or Latino patients (0.65 [0.63, 0.68]), patients of unknown race/ethnicity (0.85 [0.81, 0.90]), and patients of other races/ethnicities (0.80 [0.68, 0.93]) in the unadjusted model across all COVID waves. Similar patterns were observed within each COVID wave. After adjusting for other factors, White patients still experienced higher mortality odds compared to Black patients (0.67 [0.65, 0.70]), Hispanic patients (0.75 [0.72, 0.78]), Asian patients (0.91 [0.84, 0.99]), and patients of unknown race/ethnicity (0.90 [0.85, 0.96]) across all COVID waves. American Indian or Alaskan Native patients, Native Hawaiian and other Pacific Islanders patients, and patients of other races/ethnicities experienced the same mortality odds as White patients. Similar patterns were observed within each COVID wave.

## Discussion

Using a retrospective national cohort of 232,364 patients between April 27, 2020 and June 25, 2022 with various comorbid diseases and demographics, we have identified important shifts in severity and mechanical ventilation linked to the differing variants not previously reported. Specifically, the overall utilization of mechanical ventilation decreased from 10% during the initial wave, 8% during alpha, 12% during delta, and down to 6% in the omicron-subsequent group. The 30-day mortality rate also decreased from 11% during the delta wave to 4% during the omicron-subsequent omicron wave. This was in line with previous studies of Omicron (B.1.1.529) in southern California which showed decreased hospital admissions, ICU admission, and mortality with Omicron vs. the Delta variant [9]. Wolter et al. also found decreased hospitalization rates and clinical severity in the Omicron variant compared to the Delta variant including decreased ICU admissions, mechanical ventilation, development of acute respiratory distress syndrome, utilization of extracorporeal membrane oxygenation, or death [5]. Dobrowolska et al. showed decreased ventilation rates at 7.2% vs. 3.1% (p < 0.001) among the Omicron variant vs. Delta variant [10]. The Omicron variant has also shown lower case fatality rates compared to the Delta variant [11]. Altarawneh et al. outlined previous SARS-CoV-2 infection as providing up to 90% protection against future re-infection during the Alpha, Beta, and Delta variants, and around 60% against Omicron variants without any cases of fatality [12]. There are several possible explanations for the observed decreased severity of COVID-19 waves including the increased update of vaccination, prior infection, earlier recognition, and healthcare practices [23–27].

Previous studies have found obesity to be associated with an increased risk of mechanical ventilation use and mortality [13–19,28,29]. Recent studies, however, have shown no clear association of obesity, but rather its related comorbidities including diabetes and hypertension [30]. Similarly, more recent studies have also shown that COPD did not show worsened outcomes in invasive ventilation utilization after adjustment for other comorbidities [31]. For patients with diabetes, our results showed higher odds of mechanical ventilation throughout the initial, Alpha, and Delta variants, however, did not have any significant increase in odds in the Omicron variates. In terms of mortality, although patients with diabetes still had an overall higher risk of 30-day mortality, the subsequent Omicron wave demonstrated no significant increase in odds of 30-day mortality compared to non-diabetic patients. During the initial variants in early 2020, diabetes was studied and found to be a high-risk comorbidity for adverse COVID-19-related outcomes including higher risk of severe pneumonia attributed to elevated laboratory inflammatory markers, endothelial damage, fibrosis, thrombosis, and vasoconstriction leading to concomitant pulmonary dysfunction [29,32,33]. Studies in 2020 showed higher mortality rates and mechanical ventilation requirements among diabetic patients [34]. A study of 681 patients in 2020 showed an increased odds ratio of 3.216 [1.134, 9.120] of mechanical ventilation for patients with diabetes and independent increase in in-hospital death with an OR of 2.33 [1.7, 3.1] by multivariate logistic analysis [35]. Kim et al. studied patients by propensity score matching in 2020 and found increased odds of 1.43 [1.09, 1.87] of severe COVID-10 including utilization of tracheostomy, ICU admission, mechanical ventilation, or renal replacement therapy among patients with diabetes [19]. This suggests that other factors, possibly related to increased vaccination efforts and the decreased severity of clinical outcomes with the Omicron variant may be contributing to the overall decline in mechanical ventilation utilization and 30-day mortality among diabetic patients. This was suggested by a large study by Naouri et al. which studied the characteristics of COVID-19 based on 3 different surges between 2020 and 2022 and showed a decrease in mechanical ventilation utilization among vaccinated patients with an adjusted sub-hazard ratio (aSHR) of 0.64 [0.53–0.76] and decrease in in-hospital death with an aSHR of 0.80 [0.68–0.95] [36].

Our study also showed that patients with obesity initially and overall had higher odds of requiring mechanical ventilation; however, in the Omicron-subsequent variant, no significant differences were observed. Similarly, among patients in 2020, Simonnet et al. showed an independent increased odds (7.36 [1.63, 33.14], *p* = .02) of mechanical ventilation requirements among patients with higher stages of obesity by multivariate logistic regression, adjusting for diabetes, hypertension, and age [14]. Naouri et al. also showed a significantly higher adjusted odds ratio of 1.85 ([1.39, 2.47], *p* < 0.001) for risk of a composite outcome of all-cause mortality, or requirement of mechanical ventilation, or requirement of extracorporeal membrane oxygenation among patients with obesity [36,37]. Several other studies have noted an association of obesity with severe COVID-19 outcomes [13–18,29]. However, more recent studies by Tong et al. demonstrated an independent association of obesity-related comorbidities including hypertension and diabetes rather than obesity itself as the cause of increased ICU admissions, mechanical ventilation, and mortality after adjustment of other comorbidities [30]. Our study similarly adjusted for sex, race/ethnicity, diabetes, obesity, comorbid cardiac, comorbid liver, and comorbid respiratory disease. Our study showed an overall increased 30-day mortality for the Delta wave; however, it similarly did not show a significant difference in mortality for patients with obesity across all other waves and overall in the adjusted model. Because obesity is more prevalent in those with diabetes and cardiac disease, its impact may be difficult to determine in the presence of these common comorbid conditions.

Liver disease, assessed by either a recorded diagnosis or elevated FIB-4 index showed a higher odds ratio of mechanical ventilation compared to those without across all COVID waves in both unadjusted and adjusted models except for Omicron-subsequent which showed no significance in the adjusted model. Our studies were in line with previous studies including Vardava et al. who showed an increased odds ratio among patients with liver disease requiring mechanical ventilation with an odds ratio of 1.5 [1.443, 1.643] [17]. Previous studies have shown increased odds of requiring mechanical ventilation with a fibrosis-4 (FIB-4) score > 2.67 across all variants of COVID-19 (OR 1.81; 95% CI: [1.76, 1.86]) [8]. Increased FIB-4 was also associated with increased 30-day mortality across all variates [8]. While several large studies have identified increased mortality and need for mechanical ventilation in patients with cardiovascular disease with subsequent variants, our results in the adjusted model showed no difference in 30-day mortality rates among variants and were lower during the Delta variant [19]. One possible explanation may be that our study differed in defining characteristics of severe COVID disease. For example, our study used end points such as continuous renal replacement therapy on top of utilization of invasive ventilation, rather than only invasive ventilation [19].

Through analysis of COVID patients with respiratory disease, our results showed a lower odds ratio of requiring mechanical ventilation during the delta wave, with no significant difference otherwise. There was an increased mortality rate in all waves except Omicron in those with comorbid respiratory disease in the unadjusted model. However, after adjustment, comorbid respiratory disease was associated with increased mortality across all waves. Although early COVID-19 literature found increased risks of ICU admissions and mortality, recent studies found that after adjusting for other comorbidities, chronic obstructive pulmonary disease (COPD) did not show worsened outcomes in invasive ventilation or mortality [28,31,38]. Vardavas also found an increased odds of mechanical ventilation among patients with asthma with an OR [95% CI] of 18 [2, 35]; however, COPD showed no significant difference at 0.661 [0.268, 1.63] [17]. Mortality-wise, however, patients with respiratory disease had an increased odds of mortality with an OR of 142 [16, 339] [17].

When controlling only for race and ethnicity, White patients had a higher risk of 30-day mortality compared to Black/AA patients, Hispanics, and Asians across all COVID waves and the same mortality risk as Native Hawaii and other Pacific Islanders patients. These findings differ from prior studies which showed an increased adjusted odds ratios (aOR) of mortality in Black (1.20 [1.15, 1.25]), Hispanic patients (1.51 [1.44, 1.57]), and Native Americans compared to White patients [23]. Romano et al. showed higher proportions of hospitalization rates among Hispanic or Latino patients from May to June 2020 and December 2020 in mid-2020 [7]. Other months showed less evident differences. Similarly, Rao et al. showed a higher utilization of mechanical ventilation, a marker of disease severity among Hispanic patients [39]. It also showed lower mortality levels, possibly due to younger ages of patients [39]. Isath et al. also outlined increased mortality rates in Black, Hispanic, and Native Americans compared to White patients [29]. Our study likely did not have a large sample size of Native Americans to detect a significant difference in ventilation rates for most waves.

## Limitations

There are some important limitations. N3C, as with any large retrospective multicenter database, is subject to selection bias. Decisions of cohort criteria and variable selection can be arbitrarily made which can impact results. Potentially arbitrary decisions in data processing stage can result in significantly different cohort sizes and characteristics, introducing biases that may impact the quality of research conclusions [40]. Furthermore, we did not perform cross-validation. If our sample (and hence results) were biased by the hospitals or patients selected to be included in N3C, cross-validating from that same data source would not address their limited generalizability. In addition, our study did not include the effect of prior infection, the impact of initial and subsequent booster vaccinations, individual decisions of and efficacy of available treatments during each variant wave, variability of practice patterns and timing of ICU care, noninvasive ventilation or on mechanical ventilation utilization on mortality. Although ICU admissions can serve as a surrogate for COVID disease severity, because different hospitals may have had different thresholds for ICU admissions, we keep ICU admission variable in our models to minimize this potential bias There was also a limited sample size of Native Americans which may have affected analysis of significance. Additionally, because we limited our cohort to those with data elements to calculate a FIB-4, our results may not be generalizable to all patients with COVID-19. We also were not able to assess the long-term outcomes of COVID-19 in each wave. Lastly, the N3C is a consortia database sourced from many institutions with multiple data models that have been harmonized into the OMOP common data model through a process that could potentially introduce errors.

## Conclusion

The utilization of mechanical ventilation and 30-day mortality decreased in the Omicron variant compared to earlier variants. Patients with diabetes showed higher odds of mechanical ventilation and 30-day mortality for all variants except the newer omicron variants. Patients with obesity had no significant difference in mortality across waves except notably had an increased 30-day mortality during the Delta wave. Liver disease showed a higher odds ratio of mechanical ventilation across all COVID waves except for the omicron-subsequent wave which showed no significance.
